# Methods for the assessment of selection bias in drug safety during pregnancy studies using electronic medical data

**DOI:** 10.1002/prp2.426

**Published:** 2018-09-21

**Authors:** Mireille E. Schnitzer, Lucie Blais

**Affiliations:** ^1^ Faculté de pharmacie Université de Montréal Montreal Canada; ^2^ Hôpital du Sacré Cœur de Montréal Centre intégré universitaire de santé et de services sociaux du Nord‐de‐l’île‐de‐Montréal Montreal Canada

**Keywords:** directed acyclic graphs, drug effectiveness and safety, live birth bias, pregnancy, selection bias, sensitivity analysis, simulation study

## Abstract

Electronic health data are routinely used for population drug studies. Due to the ethical dilemma in carrying out experimental drug studies on pregnant women, the effects of medication usage during pregnancy on fetal and maternal outcomes are largely evaluated using this data collection medium. One major limitation in this type of study is the delayed inclusion of pregnancies in the cohort. For example, in the province of Quebec, Canada, a major pregnancy cohort only captured pregnancies after 20 weeks gestation. The purpose of this study was to demonstrate three methods that can be used to assess the extent of selection bias due to the delayed inclusion of pregnancies. We use causal directed acyclic graphs to explain the source of this selection bias. In an example involving a cohort of pregnant asthmatic women reconstructed from the linkage of administrative health databases from the province of Quebec, we use numerical derivations, a simulation study and a sensitivity analysis to investigate the potential for bias and loss of power due to the delayed inclusion. We find that this selection bias can be partially mitigated by controlling for variables related to (spontaneous or therapeutic) abortion and the outcome of interest. The three proposed methods allow for the pre and post hoc ascertainment of the bias. While delayed pregnancy inclusion selection bias (which includes “live birth bias”) can produce substantial bias in pregnancy drug studies, all three methods are effective at producing estimates of the size of the bias.

**Abbreviations**
DAGs: directed acyclic graphs
RAMQ: Régie de l'assurance maladie du Québec

## 1 INTRODUCTION

Due to ethical constraints, the evaluation of drug safety during pregnancy is generally restricted to observational studies, where patients are followed through time without intervention.^1^, ^2^, ^3^ While observational studies allow for long‐term follow‐up and the inclusion of a large population in a “real‐world” context, an analysis of such data may potentially be hampered by unmeasured confounding, selection bias, and improper effect definition and estimation. Ultimately, as in all study designs, the analysis of observational data requires strict assumptions and appropriate statistical methods to produce unbiased estimates of the treatment effect.^4^

Electronic health data, extracted from health system or medical insurance administrative (claims) databases, are highly desirable due to their availability and coverage of large portions of the target population within countries’ administrative divisions.^5^ Such data are often used to investigate drug safety during pregnancy and there has been much discussion of methodological considerations in this setting.^6^, ^7^, ^8^ In particular, as these data were not collected for research purposes, health conditions, and medications may be only partially observed. In particular, pregnancy status may only be recorded after crossing a gestational time threshold.^9^, ^10^, ^11^, ^12^ For example, depending on the data source, pregnancy cohorts may be composed exclusively of live births^13^, ^14^, ^15^ or pregnancies that survive past a certain threshold. We refer to such situations as the delayed inclusion of pregnancies in the cohort.

We describe an example from the province of Quebec, Canada that aims to evaluate the safety of similarly indicated asthma medications on pregnancy outcomes. The usage of electronic health data in this example relies on an extraction of provincial medical insurance data where the cohort is defined in terms of “deliveries,” that is, all pregnancies that surpass a 20 week threshold.^16^ We use directed acyclic graphs (DAGs)^17^ to demonstrate that the delayed inclusion of pregnancies can lead to bias in the estimation of drug safety or effectiveness. Such bias is often termed “selection” or “collider” bias and can often not be removed using the restricted cohort and limited measured information.^18^, ^19^, ^20^ However, one can evaluate the possible extent of the bias by imputing plausible values for several inestimable associations.^20^ We therefore describe three strategies to evaluate the potential impact of the selection bias in this setting: (1) numerical derivations, which plot the resulting level of bias conditional on a plausible range of associations, (2) simulation studies, which require fixing single values for the unobservable associations, and (3) post hoc sensitivity analysis to determine whether selection bias, under plausible assumptions, could have affected the statistical conclusions of the study. Finally, the code to implement the numerical derivation and simulation study are available in the Web Appendix.

## 2 EXAMPLE: THE EFFECT OF ASTHMA MEDICATION DURING PREGNANCY

While many of the principles evaluated in this article apply in a large range of settings, we focus on the safety of asthma medication taken by pregnant asthmatic women in a cohort of pregnancies. Current guidelines suggest that pregnant women suffering from asthma continue their standard treatment throughout pregnancy due to the dangers of uncontrolled asthma provoked by stopping therapy.^21^ However, interest lies in the relative effects of different treatment options and intensities on various outcomes related to the fetus and maternal health.

The Québec Asthma and Pregnancy Database^22^ was obtained through a linkage of the Régie de l'assurance maladie du Québec (RAMQ) and the MED‐ECHO databases. RAMQ, the universal health care system in the province of Québec, Canada, defines delivery as all live or stillbirths occurring after the first completed 20 weeks of pregnancy. The data extraction took all deliveries between the years 1990 and 2010 for women ≤45 years with at least one asthma diagnosis in the 2 years prior to delivery and a random sample of other pregnant women. For inclusion, these women also had to be covered by the Québec public drug insurance plan in the year prior to and during pregnancy. Eltonsy et al^22^ contrasted treatment options for different asthma severity levels on major congenital malformations recorded at birth or during the first year of life. The outcome was identified using codes from the International Classification of Diseases ninth and tenth revisions with more details provided in the original manuscript. In women with moderate asthma, they compared two alternative treatments over the span of the first trimester: (1) a low dose of inhaled corticosteroids plus the add‐on therapy of long‐acting β_2_‐agonists vs (2) a higher dose of inhaled corticosteroids and no add‐on therapy. The exposure was measured over the first trimester (ie, the first 12 weeks of gestation) due to their hypothesis that this corresponds with a teratogenic window, as discussed in the original manuscript.

The investigations in this manuscript did not involve individual patient data and the study is therefore exempt from institutional ethics review.

## 3 A DIRECTED ACYCLIC GRAPH

**Figure 1:**
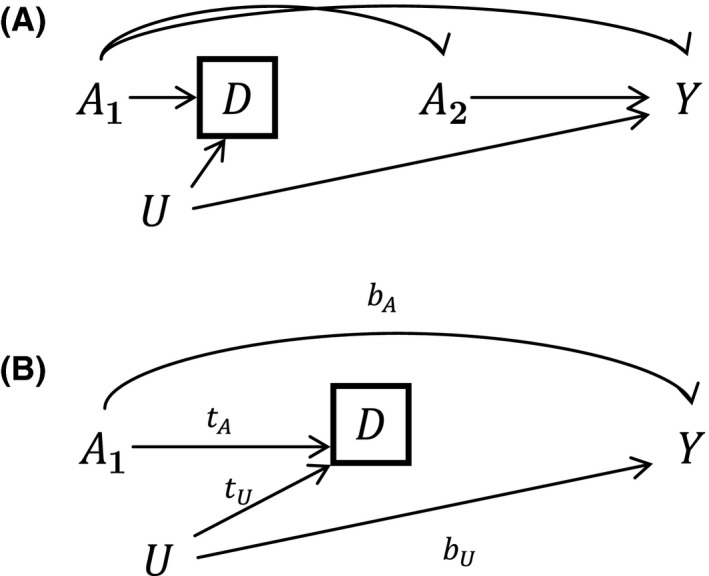
Collider Bias in Delivery Cohorts. D represents delivery, defined as birth after 20 weeks. *A*_1_ is the exposure to the medication before 20 weeks and *A*_2_ is exposure after 20 weeks

The identification and selection of subjects into an analysis can produce bias in the effect estimation due to selection on a collider variable.^19^ In Figure 1 we present two examples of selection due to the definition of “delivery” in the RAMQ administrative health database; pregnancies are only classified as deliveries (and available in our cohort) if they surpass the 20 week mark. It is estimated that 13%‐15% of pregnancies end in spontaneous abortion^23^ and 21% in induced abortion (in Canada and the United States)^24^ and it is thought that these numbers may be underestimated,^25^ so this selection is not trivial.

The arrows (or directed edges) between variables indicate that one variable (the *parent*) affects another (the *child*) in the direction of the arrow. The dotted lines indicate correlations between two variables due to latent and temporally prior variables. A *path* between two variables is an unbroken route that proceeds along or against the direction of the arrows. A path is considered open unless (1) conditioning on a variable blocks the path (denoted by a square around the variable) or (2) the path goes through an unadjusted *collider*: a variable that is affected by two parent variables. Adjusting for a collider opens the previously blocked path. If there is an open path between the outcome and exposure other than the path of interest, estimation of the causal effect will be biased.^26^

In the DAG in Figure 1, we consider a scenario where an investigator is interested in estimating the effect of exposure to a medication during early pregnancy on a birth outcome. In our example, this corresponds to early usage of asthma controller medications in the first trimester ($A_{1}$) and major congenital malformations (Y) which can occur at any time during gestation. We consider the setting where the early medication exposure ($A_{1}$) may result in an increased risk of abortion before 20 weeks.^27^ D represents the classification as a delivery by the RAMQ. It is possible that there exist unmeasured variables U that affect (spontaneous or therapeutic) abortion before 20 weeks and the outcome (congenital malformations), such as smoking and socio‐economic factors.^10^ For simplicity, confounders of exposure and outcome are assumed measured and adjusted for and do not appear in the DAG, and while U may be time‐varying or multidimensional, we group it into a single variable. Stratification by D, which is a collider of the variables $A_{1}$ and U, creates a correlation between these two latter variables, opening up a path between $A_{1}$ and the outcome Y. This causes confounding of the early exposure ($A_{1}$) and outcome association. The magnitude of the bias will depend on the strength of the effect of the unmeasured variable U on abortion and outcome. This bias should be considered in any pregnancy cohort where failed or terminated pregnancies are not included. The effect of average exposure over the entire duration of the pregnancy may also be confounded.

If U cannot be controlled for in the analysis, one option is to change the question of interest to investigate the effect of exposure to medication past 20 weeks ($A_{2}$) on the birth outcome, while adjusting for $A_{1}$ in order to close all backdoor paths. However, this is hardly satisfactory when early exposure is believed to be responsible for a specific birth outcome, such as for congenital malformations.^22^ In addition, the estimation of the effect of $A_{2}$ would require that many women changed their treatment categories over the two time points (otherwise the effect of later exposure would be entirely confounded with that of $A_{1}$). Other options are to explore the extent of the bias using numerical derivations, simulation studies, and sensitivity analyses as we do in the next section.

A structurally equivalent type of bias has been shown to arise in cohorts defined by an index event such as disease occurrence when assessing the association between exposure to medication and an outcome such as mortality.^28^, ^29^ This *index event bias* has been known to at times invert the association between the exposure of interest and the outcome; for example, smoking has been shown to be protective of subsequent myocardial infarction in cohorts of patients who had a first myocardial infarction, while a harmful effect of smoking is biologically plausible.^30^ In another example, index event bias was shown to explain the apparent protective effect of obesity on mortality in patients with cardiovascular disease.^31^

## 4 IMPACT OF POSTEXPOSURE SELECTION ON BIAS AND STATISTICAL POWER

While there are many sources of bias in epidemiologic studies, the particularities of the data determine whether the bias has an important impact on the scientific conclusions. Consider the example concerning selection on births past 20 weeks. A simplified DAG is presented in Figure 1B where $A_{1}$ is an asthma medication in the first trimester of pregnancy and the outcome Y is major congenital malformations.

We also only consider a univariate U in the following development, though additional complexities may be added with modifications to the code. An example of such a variable (U) is antidepressant medication taken during pregnancy. This variable was not assessed and therefore not adjusted for in the Eltonsy study, making it a potential source of some selection bias. There is observational study evidence of impacts of antidepressant medications on spontaneous abortion with odds ratios between 1.1 and 1.7^32^, ^33^ and on major malformations with odds ratios between 1 and 3^34^, ^35^, ^36^ though meta‐analysis concluded that associations only appear to be present for cardiac malformations.^35^ Using these estimates to inform our sensitivity analyses, we are assuming that these estimated effects on spontaneous abortions correspond to the effects on all abortion and that the effects in the general population correspond to the effects in asthmatic women. In order to express the uncertainty in these estimates, we investigate an extended range of possible effect sizes. We now describe three methods to evaluate the potential selection bias for various values of these associations.

### 4.1 Numerical example

**Figure 2:**
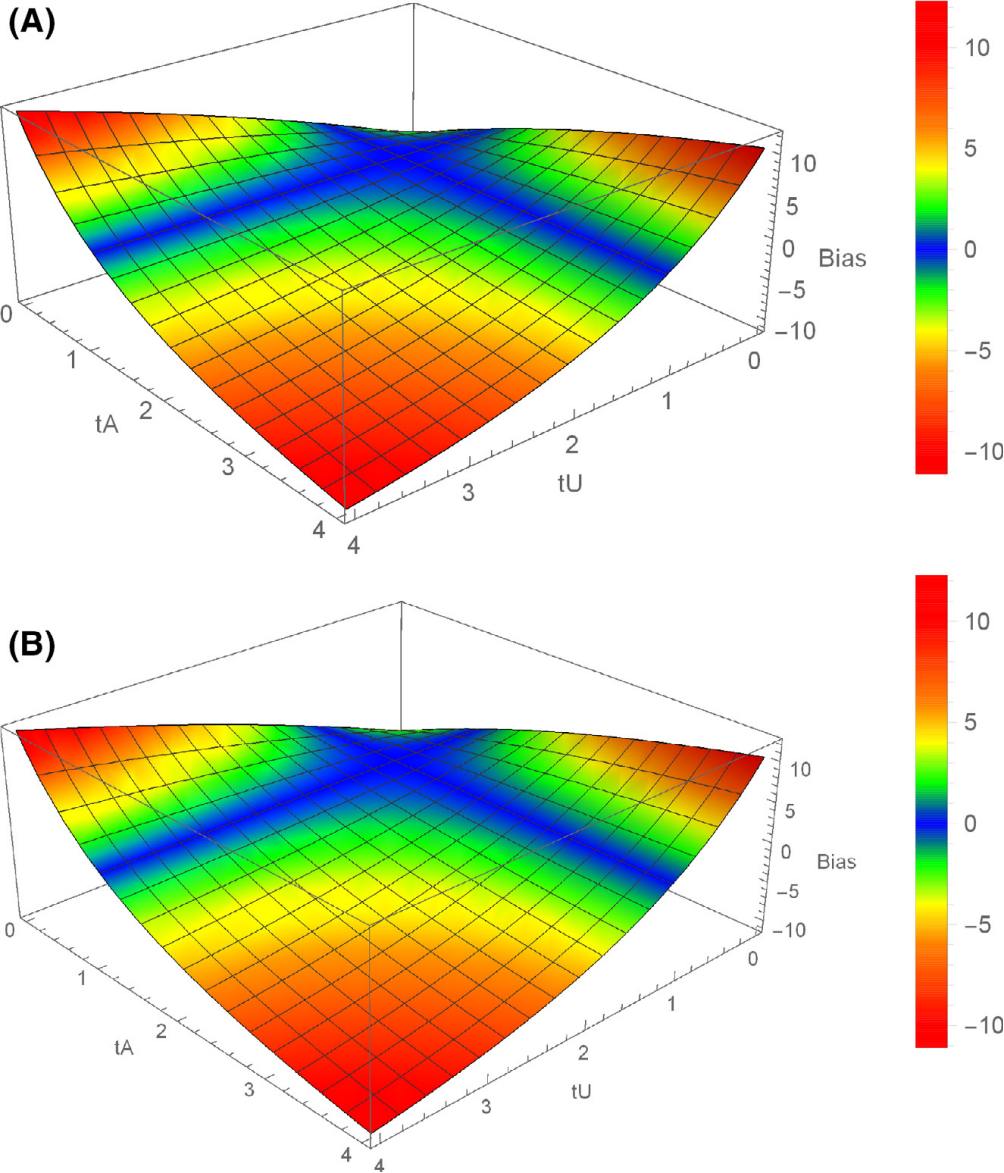
% True Bias in the Odds Ratio Caused by Selection on Deliveries in the Numerical Example. % bias = (conditional/true − 1)*100 when the true exposure effect odds ratio is (A) 1 and (B) 1.3. Note the absence of bias when *t*_U_ = 1 or *t*_A_ = 1, that is, when *D* is not a collider

By specifying the distributions and relationships between the covariates in the DAG of Figure 1B, we can calculate the exact bias caused by the selection on deliveries. To this end, we assume that the variables are generated on a logit‐linear scale with associations parameterized by conditional odds ratios. In particular, $b_{A}$ represents the effect size (conditional odds ratio) of exposure on outcome, while $b_{U}$ represents the effect of the unmeasured variable U on outcome. The parameters $t_{A}$ and $t_{U}$ represent the effects of A and U, respectively, on the probability of the pregnancy surviving the 20th week. We suppose that the baseline risks of abortion and gestational malformations are about 18% and 8%,^22^ respectively, but that these risks can be exacerbated by a binary U. Letting the true effect size be $b_{A}=1$ (no effect) and 1.3, respectively, and setting $b_{U}=3$ (strong effect of U on outcome), the bias in the marginal effect that conditions on deliveries compared to the true marginal effect is given in 3D graphics in Figure 2. Bias in the conditional odds ratio is defined as (conditional/true−1)×100. Even for large associations $t_{A}$ and $t_{U}$, the bias in our example remains fairly small, dropping to −10% only when being exposed and having U=1 both lead to a 4‐fold increase in the odds of delivery over the baseline and when the true odds ratio for the effect of interest is 1.3. These results suggest that the biased analyses would be estimating odds ratios of 0.9 if there is no effect or roughly 1.2 if the true effect corresponds to an odds ratio of 1.3. While the bias is proportionally small, with sufficient data this could result in different scientific conclusions. Attenuating the association $b_{U}$ results in a reduction in bias (results given in Appendix A2).

A strength of this approach is that one can visually investigate the trends in bias while modifying the values of two parameters at a time. It is also possible to increase the complexity of the assumed DAG, for instance, by considering the two distinct types of pregnancy loss or having multiple U variables (although this will also create new parameters to either assign values to or vary over a range of possible values). One weakness of this approach is the analyst must assign specifications for the distributions of Y and D and that the results may vary depending on this specification. In our example, we assumed that the probabilities of these binary variables are generated on the logit‐linear scale, conditional on the prior variables.

In the Web Appendix A1, we provide the Mathematica (Wolfram Research, Inc, Champaign, Illinois, USA) code used to produce the graphics in Figure 2.

### 4.2 Simulation study

The 3D graphic allowed us to observe how the selection bias varies continuously with the importance of the unmeasured variable U. Using the same data generating assumptions as in the numerical example and setting a range of values for the parameters $t_{A}$, $t_{U}$, $b_{A}$, and $b_{U}$, we can alternatively perform a Monte Carlo simulation study to estimate the expected bias and power to detect an effect of A on Y. We generated 1000 datasets each one representing N=10000 pregnancies subject to selection on delivery with the same baseline odds of abortion and gestational malformations (18% and 8%, respectively). For each dataset, we calculated the odds ratio for the effect of interest using (1) only deliveries and (2) a random subset of pregnancies of the same number (which emulates a setting without selection due to abortion with the same sample size). We look at both bias and power to detect an effect over a wide range of associations in Table 1. We test three small effect sizes: odds ratios of 1.1, 1.2, and 1.3. Corresponding to Figure 2, the bias remains small except in the most extreme cases where the bias reached −7.7%. However, the power to detect an effect can be drastically reduced by the selection on D=1 compared to random selection. We see the largest effects on power when the study is just barely well‐powered or under‐powered. For example, when $t_{A}=2$ and $t_{U}=b_{U}=3$ and the true effect was 1.2, the selection on a collider reduced the power from 68% to 42%. In the most extreme case ($t_{A}=t_{U}=b_{U}=3$) with the smallest effect size, power was four times greater without collider bias.

**Table 1:** Percent bias and (in brackets) percent of significant (*P* < 0.05) associations in a simulation study with 1000 random generations of N=10000 pregnancies

		$t_{A}$:		1 (D is not a collider)		2		3	
		cOR ($b_{A}$) (true effect)		D = 1	Random	D = 1	Random	D = 1	Random
Weak risk factor	$t_{U}$= 1.5 $b_{U}$= 1.5	1.1		0 (22)	0 (24)	−0.9 (19)	0 (21)	0 (18)	0 (19)
		1.2		0 (65)	0 (67)	−0.8 (62)	0 (61)	0 (53)	0 (50)
		1.3		0 (93)	0 (94)	−0.8 (90)	0 (90)	0 (85)	0 (84)
Moderate risk factor	$t_{U}$= 2 $b_{U}$= 2	1.1		0 (22)	0 (22)	−1.8 (16)	0 (20)	−2.7 (14)	0 (20)
		1.2		−0.8 (67)	−0.8 (67)	−1.7 (55)	0 (64)	−3.3 (48)	0 (59)
		1.3		−0.8 (95)	−0.8 (95)	−2.3 (90)	0 (94)	−3.1 (84)	0 (90)
Strong risk factor	$t_{U}$= 3 $b_{U}$= 3	1.1		0 (26)	0 (26)	−4.5 (10)	0 (23)	−7.3 (6)	0 (23)
		1.2		0 (72)	0 (77)	−5.0 (42)	0 (68)	−7.5 (27)	0 (64)
		1.3		−0.8 (95)	0 (97)	−5.4 (79)	0 (94)	−7.7 (64)	0 (93)

We contrast signal detection with selection on pregnancies past 20 weeks (*D = 1*) vs random selection of the same number of subjects (Random). All parameters used in the data generation (*b*_A_, *t*_A_, *t*_U_, and $b_{U}$) are expressed as odds ratios.

While the bias in this example is relatively small, it is highly dependent on the baseline risks of outcome and selection. When increasing both baseline risks to 50%, the maximum bias increased to 25%.

One strength of this approach is that, unlike for the numerical example, one can vary multiple parameters in the same table; we varied four parameters in Table 1. As in the numerical example, one can also increase the complexity of the DAG. A particular advantage of the simulation study is that one can investigate the estimation bias, standard error, and power of the statistical estimator, while the numerical example only compares the bias in the conditional odds ratio (ie, the bias in what one would estimate with infinite data). The requirement of making arbitrary distributional assumptions is also a limitation of this approach.

In the Web Appendix A3, we provide the R software (https://www.r-project.org/) code used in this simulation study.

### 4.3 Sensitivity analysis

Sensitivity analysis can be used to evaluate the potential impact of selection bias on the scientific conclusion of a given study. Banack and Kaufman^31^ demonstrate how sensitivity analysis for mediation^37^ can be used to evaluate the impact of index‐event bias. Starting from estimates obtained in a real study, we evaluate the potential for bias due to selection on births past 20 weeks. In Eltonsy et al^22^ exposure to long‐acting β_2_‐agonists and inhaled corticosteroids was assessed during the first trimester of pregnancy. In women with moderate asthma, the contrast of interest was the relative effect of low‐dose inhaled corticosteroids plus long‐acting β_2_‐agonists vs medium‐dose inhaled corticosteroids therapy on the risk of major congenital malformation. This analysis did not adjust for variables that may cause both pregnancy loss before 20 weeks and the outcome as this type of selection bias was not noted at the time.

Corresponding with parameter $b_{A}$ in Figure 1B, the true causal effect of medication on the outcome in deliveries is analogous to a controlled direct effect with a mediator D confounded by the unobserved U. If U is a single binary variable, such as antidepressant use, and assuming that all confounders of the exposure‐outcome are measured and are not caused by nor cause U, sensitivity analysis can be performed using the bias formula presented in VanderWeele.^37^ Suppose the parameter of interest is defined as the conditional risk ratio in mean outcomes amongst deliveries for exposed vs unexposed women. Letting C be all confounders of the association between A and Y, the correction factor for the estimated risk ratio can be given as follows:

$${\text{CF}}_{C}^{\text{RR}}=\frac{1+\left(\gamma -1\right)\sigma \left(A=1\right)}{1+\left(\gamma -1\right)\sigma \left(A=0\right)}$$

where $\gamma =P\left(Y=1|A,D=1,C=c,U=1\right)/P\left(Y=1|A,D=1,C=c,U=0\right)$ and $\sigma \left(A=a\right)=P\left(U=1|A=a,D=1,C\right)$. In the example, the sensitivity parameter γ can be interpreted as the conditional risk ratio for antidepressant users vs nonusers, and $\sigma \left(A=a\right)$ can be interpreted as the conditional probability of antidepressant use under exposure a.

Investigators might demonstrate the sensitivity of the estimate to the most extreme plausible values for these three sensitivity parameters. For simple usage of this bias formula, it must be assumed that γ is constant for all levels of C and A and that $\sigma \left(A=a\right)$ is constant over levels of C. Then, the risk ratio estimate and confidence interval can be corrected by multiplying by the correction factor. For rare outcomes, this procedure can also be used to perform a sensitivity analysis for the estimated odds ratios. In Eltonsy et al^22^ the adjusted odds ratio for the association between therapy choice and the risk of congenital malformation was 1.1 with 95% confidence interval: 0.6‐1.9. Given that the rate of anti‐depressant usage during pregnancy was estimated to be about 13%,^38^, ^39^ we consider extreme possibilities of $\sigma \left(A=1\right)=0.25$ and $\sigma \left(A=0\right)=0.1$. In order to raise the lower bound of the confidence interval to 1.0 (and thus conclude a significant effect), the outcome risk ratio γ for exposure to anti‐depressants would have to be at least 9. In this case, ${\text{CF}}_{C}^{\text{RR}}=5/3$ and the maximum sensitivity bound for the estimate would be 1.8 with 95% confidence interval: 1.0‐3.2. Given that such a high value for γ would indicate an unrealistically large increase in risk of induced and spontaneous abortions, it is implausible that selection bias has masked a difference in safety between the two asthma therapy options contrasted. However, due to the low power (wide confidence intervals) of the original results, this still does not provide substantial evidence that an effect does not exist.

Alternative bias formulas exist for settings in which U may also affect the exposure, though these are far less simple than the ones above.^37^ An additional limitation of this approach is that only one binary variable U may be considered at a time. This is a limitation because while one variable (such as anti‐depressant use) may not produce enough bias to create a misleading effect estimate, multiple variables (such as anti‐depressant use, smoking, and socio‐economic status) combined may have a greater impact.

## 5 DISCUSSION

In this article, we demonstrated that selection bias in electronic medical data may arise when defining the cohort on a postexposure variable such as limiting inclusion to pregnancies that pass a certain time threshold. This includes the bias that arises from selecting only on live births, since this outcome occurs after the exposure to medication during pregnancy. Recent work demonstrated the potential for bias in the estimation of exposure effects when selecting on live births in settings where exposure contributes to the abortion of fetuses.^14^, ^15^, ^40^ Additional work demonstrated the potential for bias and accuracy loss in a very similar setting where left‐truncation is differential by exposure group.^12^ We extend this work by demonstrating strategies that allow the investigator to ascertain the potential impact of selection on the scientific conclusions. Indeed, we found that the magnitude of the bias depends on the particularities of the data, including the baseline risks of the outcome and selection, emphasizing the need for study‐specific bias assessment.

In standard observational studies, investigators will typically only consider adjusting for suspected confounders between the exposure and outcome. Recognizing selection bias should motivate investigators to attempt to measure and adjust for a wider set of covariates, potentially mitigating this additional source of bias. If the additional variables are unavailable, we demonstrated how it is possible to investigate the potential for bias in a given situation. We also showed how one might alternatively assess the sensitivity of the estimated effect to different strengths of selection bias.

In addition to the single source of postexposure selection that we investigated in this study, fetal death beyond 20 weeks may pose an additional source of selection bias if these pregnancies are not included in the analysis. If the outcome of interest is undefined for nondeliveries or fetal deaths, then standard methods to estimate population average causal effects no longer apply. Under additional assumptions and external information, one may alternatively estimate the survivor average causal effect,^41^ corresponding with the effect of exposure in pregnancies that would always have lasted past 20 weeks and had a defined outcome regardless of the exposure received. However, this topic is beyond the scope of the current article.

Not all sources of bias in epidemiological studies threaten the overall validity of the conclusions; it is important to investigate the potential size of bias in relation to effect estimates. A greater understanding of the mechanics of statistical association can also guide attempts to reduce estimation bias. These pursuits will lead to more reliable studies and more nuanced conclusions of causal effects.

## DISCLOSURE

This paper considers methodological approaches to data analysis and bias. MES and LB have no conflicts of interest to report. No data were collected for this study. All data were simulated or obtained in aggregate from existing publications.

## References

[1] Koren G , Pastuszak A , Ito S . Drugs in pregnancy. N Engl J Med. 1998;338:1128‐1137. 9545362 10.1056/NEJM199804163381607

[2] Addis A , Sharabi S , Bonati M . Risk classification systems for drug use during pregnancy: are they a reliable source of information? Drug Saf. 2000;23:245‐253. 11005706 10.2165/00002018-200023030-00006

[3] Margulis AV , Andrews EB . The Safety of Medications in Pregnant Women: An Opportunity to Use Database Studies. Pediatrics. 2017;140: pii: e20164194. 28759401 10.1542/peds.2016-4194

[4] Rubin DB . ‘For objective causal inference design trumps analysis’. Ann Appl Stat. 2008;2:808‐840.

[5] Schneeweiss S . Learning from big health care data. N Engl J Med. 2014;370:2161‐2163. 24897079 10.1056/NEJMp1401111

[6] Margulis AV , Palmsten K , Andrade SE , et al. Beginning and duration of pregnancy in automated health care databases: review of estimation methods and validation results. Pharmacoepidemiol Drug Saf. 2015;24:335‐342. 25627986 10.1002/pds.3743

[7] Swanson SA , Hernandez‐Diaz S , Palmsten K , Mogun H , Olfson M , Huybrechts KF . Methodological considerations in assessing the effectiveness of antidepressant medication continuation during pregnancy using administrative data. Pharmacoepidemiol Drug Saf. 2015;24:934‐942. 26045370 10.1002/pds.3798 PMC4822692

[8] Palmsten K , Huybrechts KF , Kowal MK , Mogun H , Hernandez‐Diaz S . Validity of maternal and infant outcomes within nationwide Medicaid data. Pharmacoepidemiol Drug Saf. 2014;23:646‐655. 24740606 10.1002/pds.3627 PMC4205050

[9] Andrade SE , Scott PE , Davis RL , et al. Validity of health plan and birth certificate data for pregnancy research. Pharmacoepidemiol Drug Saf. 2013;22:7‐15. 22753079 10.1002/pds.3319 PMC3492503

[10] Cea‐Soriano L , Rodriguez LAG , Cantero OF , Hernandez‐Diaz S . Challenges of using primary care electronic medical records in the UK to study medications in pregnancy. Pharmacoepidemiol Drug Saf. 2013;22:977‐985. 23813653 10.1002/pds.3472

[11] Joly MP , Boivin M , Junker A , Bocking A , Kramer MS , Atkinson SA . An inventory of Canadian pregnancy and birth cohort studies: research in progress. BMC Pregnancy Childbirth. 2012;12:117. 23101595 10.1186/1471-2393-12-117 PMC3542086

[12] Schisterman EF , Cole SR , Ye A , Platt RW . Accuracy loss due to selection bias in cohort studies with left truncation. Paediatr Perinat Epidemiol. 2013;27:491‐502. 23930785 10.1111/ppe.12073 PMC6151356

[13] Evans DJ , Levene MI . Evidence of selection bias in preterm survival studies: a systematic review. Arch Dis Child Fetal Neonatal Ed. 2001;84:F79‐F84. 11207220 10.1136/fn.84.2.F79 PMC1721223

[14] Levy A , Matok I , Gorodischer R , et al. Bias toward the null hypothesis in pregnancy drug studies that do not include data on medical terminations of pregnancy: the folic acid antagonists. J Clin Pharmacol. 2012;52:78‐83. 21343345 10.1177/0091270010390806

[15] Liew Z , Olsen J , Cui X , Ritz B , Arah OA . Bias from conditioning on live birth in pregnancy cohorts: an illustration based on neurodevelopment in children after prenatal exposure to organic pollutants. Int J Epidemiol. 2015;44:345‐354. 25604449 10.1093/ije/dyu249 PMC4339763

[16] Blais L , Beauchesne MF , Rey E , Malo JL , Forget A . Use of inhaled corticosteroids during the first trimester of pregnancy and the risk of congenital malformations among women with asthma. Thorax. 2007;62:320‐328. 17121872 10.1136/thx.2006.062950 PMC2092465

[17] Pearl J . Causality: models, Reasoning, and Inference (Cambridge University Press). Cambridge: United Kingdom; 2009.

[18] Greenland S . Basic methods for sensitivity analysis of biases. Int J Epidemiol. 1996;25:1107‐1116. 9027513

[19] Hernan MA , Hernandez‐Diaz S , Robins JM . A structural approach to selection bias. Epidemiology. 2004;15:615‐625. 15308962 10.1097/01.ede.0000135174.63482.43

[20] Greenland S . Quantifying biases in causal models: classical confounding vs collider‐stratification bias. Epidemiology. 2003;14:300‐306. 12859030

[21] Lim A , Stewart K , Abramson MJ , Walker SP , George J . Multidisciplinary approach to management of maternal asthma (MAMMA [copyright]): the PROTOCOL for a randomized controlled trial. BMC Public Health. 2012;12:1094. 23253481 10.1186/1471-2458-12-1094 PMC3536559

[22] Eltonsy S , Forget A , Beauchesne MF , Blais L . Risk of congenital malformations for asthmatic pregnant women using a long‐acting beta(2)‐agonist and inhaled corticosteroid combination versus higher‐dose inhaled corticosteroid monotherapy. J Allergy Clin Immunol. 2015;135:123‐130. 25226849 10.1016/j.jaci.2014.07.051

[23] Goldhaber MK , Fireman BH . The fetal life table revisited: spontaneous abortion rates in three Kaiser Permanente cohorts. Epidemiology. 1991;2:33‐39. 2021664

[24] Sedgh G , Henshaw S , Singh S , Ahman E , Shah IH . Induced abortion: estimated rates and trends worldwide. Lancet. 2007;370:1338‐1345. 17933648 10.1016/S0140-6736(07)61575-X

[25] Jones RK , Kost K . Underreporting of induced and spontaneous abortion in the United States: an analysis of the 2002 National Survey of Family Growth. Stud Fam Plann. 2007;38:187‐197. 17933292 10.1111/j.1728-4465.2007.00130.x

[26] Greenland S , Pearl J , Robins JM . Causal diagrams for epidemiologic research. Epidemiology. 1999;10:37‐48. 9888278

[27] Blais L , Kettani FZ , Forget A . Relationship between maternal asthma, its severity and control and abortion. Hum Reprod. 2013;28:908‐915. 23427230 10.1093/humrep/det024

[28] Dahabreh IJ , Kent DM . Index event bias as an explanation for the paradoxes of recurrence risk research. JAMA. 2011;305:822‐823. 21343582 10.1001/jama.2011.163 PMC4115579

[29] Smits LJ , van Kuijk SM , Leffers P , Peeters LL , Prins MH , Sep SJ . ‘Index event bias‐a numerical example’. J Clin Epidemiol 2013;66:192‐196. 23257150 10.1016/j.jclinepi.2012.06.023

[30] Barbash GI , Reiner J , White HD , et al. ‘Evaluation of paradoxic beneficial effects of smoking in patients receiving thrombolytic therapy for acute myocardial infarction: mechanism of the “smoker's paradox” from the GUSTO‐I trial, with angiographic insights. Global Utilization of Streptokinase and Tissue‐Plasminogen Activator for Occluded Coronary Arteries’. J Am Coll Cardiol. 1995;26:1222‐1229. 7594035 10.1016/0735-1097(95)00299-5

[31] Banack HR , Kaufman JS . Does selection bias explain the obesity paradox among individuals with cardiovascular disease? Ann Epidemiol. 2015;25:342‐349. 25867852 10.1016/j.annepidem.2015.02.008

[32] Nakhai‐Pour HR , Broy P , Berard A . Use of antidepressants during pregnancy and the risk of spontaneous abortion. CMAJ. 2010;182:1031‐1037. 20513781 10.1503/cmaj.091208 PMC2900326

[33] Kjaersgaard MI , Parner ET , Vestergaard M , et al. Prenatal antidepressant exposure and risk of spontaneous abortion ‐ a population‐based study. PLoS ONE. 2013;8:e72095. 24015208 10.1371/journal.pone.0072095 PMC3756033

[34] Berard A , Zhao JP , Sheehy O . Antidepressant use during pregnancy and the risk of major congenital malformations in a cohort of depressed pregnant women: an updated analysis of the Quebec Pregnancy Cohort. BMJ Open. 2017;7:e013372. 10.1136/bmjopen-2016-013372 PMC5278249 28082367

[35] Grigoriadis S , VonderPorten EH , Mamisashvili L , et al. ‘Antidepressant exposure during pregnancy and congenital malformations: is there an association? A systematic review and meta‐analysis of the best evidence’ J Clin Psychiatry. 2013;74:e293‐e308. 23656855 10.4088/JCP.12r07966

[36] ‘SSRI antidepressants and birth defects’. Prescrire Int 2006;15:222‐223. 17167929

[37] VanderWeele TJ . Bias formulas for sensitivity analysis for direct and indirect effects. Epidemiology. 2010;21:540‐551. 20479643 10.1097/EDE.0b013e3181df191c PMC4231822

[38] Al‐Sahab B , Saqib M , Hauser G , Tamim H . Prevalence of smoking during pregnancy and associated risk factors among Canadian women: a national survey. BMC Pregnancy Childbirth. 2010;10:24. 20497553 10.1186/1471-2393-10-24 PMC2885995

[39] Cooper WO , Willy ME , Pont SJ , Ray WA . Increasing use of antidepressants in pregnancy. Am J Obstet Gynecol. 2007;196(544):e1‐e5. 10.1016/j.ajog.2007.01.033 17547888

[40] Margulis AV , Mittleman MA , Glynn RJ , Holmes LB , Hernandez‐Diaz S . Effects of gestational age at enrollment in pregnancy exposure registries. Pharmacoepidemiol Drug Saf. 2015;24:343‐352. 25702683 10.1002/pds.3731

[41] Tchetgen Tchetgen EJ . Identification and estimation of survivor average causal effects. Stat Med. 2014;33:3601‐3628. 24889022 10.1002/sim.6181 PMC4131726

