# Supplementary material for: Methods for the assessment of selection bias in drug safety during pregnancy studies using electronic medical data
Source: Pharmacol Res Perspect. 2018 Sep 21;6(5):e00426. doi: 10.1002/prp2.426 (PMC6149369; doi:10.1002/prp2.426)
Supplement: Supplementary file 1 [file PRP2-6-e00426-s001.docx]

**WEB APPENDIX**

**A1. Numerical example Mathematica (Wolfram Research, Inc, Champaign, Illinois, USA) code**

This code creates the bias colour scheme used in the Figure 2.

new={{"Bias11","",{}}, {"Physical"}, 1, {-10,10}, {{-10.,Red}, {-6.,Orange}, {-4,Yellow},{-2,Green}, {0.,Blue}, {2,Green}, {4.,Yellow}, {6.,Orange}, {10.,Red}}, ""}

AppendTo[DataPaclets`ColorDataDump`colorSchemes,new];

AppendTo[DataPaclets`ColorDataDump`colorSchemeNames,new[[1,1]]];

**A1.1 Code corresponding to the results in the paper.**

The following code produces the 3D graphs in Figure 2. As written, it includes a harmful effect of A on Y (OR=1.3) but this can be changed to 1 for a null effect.

ClearAll["Global`*"]

expit[x_] := 1/(1 + Exp[-x])

b0 = -2.5 (* controls the baseline risk of outcome *)

pY1[U_]:=expit[b0+bU*U+Log[1.3]]

pY0[U_]:=expit[b0+bU*U]

pS1[U_]:=expit[t0+tU*U+tA] (* probability of miscarriage *)

pS0[U_]:=expit[t0+tU*U]

(* reparametrize to express tU and tA in OR*)

tU=Log[tauU]

tA=Log[tauA]

t0=-1.5 (* controls the baseline risk of non-selection (e.g. miscarriage) *)

(* this implies that without other risk factors, the baseline risk of non-selection is expit(-1.5) *)

bU=Log[3] (* controls the effect of U on outcome; here, OR=3 *)

(* calculate effect conditioning on delivery *)

EY1D1=(pY1[1]*(1-pS1[1])*0.5+pY1[0]*(1-pS1[0])*0.5)/((1-pS1[1])*0.5+(1-pS1[0])*0.5)

EY0D1=(pY0[1]*(1-pS0[1])*0.5+pY0[0]*(1-pS0[0])*0.5)/((1-pS0[1])*0.5+(1-pS0[0])*0.5)

(* calculate true effect *)

EY1=pY1[1]*0.5+pY1[0]*0.5

EY0=pY0[1]*0.5+pY0[0]*0.5

Plot3D[((((EY1D1/(1-EY1D1))/(EY0D1/(1-EY0D1)))/((EY1/(1-EY1))/(EY0/(1-EY0))))-1)*100, {tauU,0,4}, {tauA,0,4}, AxesLabel->{"tU","tA","Bias"}, ColorFunction->ColorData["Bias11"], ColorFunctionScaling->False, PlotLegends->Automatic]

**A1.2 Code corresponding to the results presented in A2**

The following code allows for the visualization of the bias changes caused by varying $b_{U}$ (and can be run after the previous code segments). Here, we use a null effect of A on Y though this can be modified as before. The resulting graph is given in the next section of the Appendix.

Clear[bU]

tA = Log[2]

tauA = Exp[tA]

b0 = -2.5

pY1[U_] := expit[b0 + bU*U]

pY0[U_] := expit[b0 + bU*U]

pS1[U_] := expit[t0 + tU*U + tA] (* prob of miscarriage *)

pS0[U_] := expit[t0 + tU*U]

(*reparametrize*)

tU = Log[tauU]

bU = Log[tauB]

t0 = -1.5

EY1D1 = (pY1[1]*(1 - pS1[1])*0.5 +

pY1[0]*(1 - pS1[0])*0.5)/((1 - pS1[1])*0.5 + (1 -

pS1[0])*0.5) (*conditioning on delivery*)

EY0D1 = (pY0[1]*(1 - pS0[1])*0.5 +

pY0[0]*(1 - pS0[0])*0.5)/((1 - pS0[1])*0.5 + (1 - pS0[0])*0.5)

(*true effect*)

EY1 = pY1[1]*0.5 + pY1[0]*0.5

EY0 = pY0[1]*0.5 + pY0[0]*0.5

Plot3D[((((EY1D1/(1 - EY1D1))/(EY0D1/(1 - EY0D1)))/((EY1/(1 -

EY1))/(EY0/(1 - EY0)))) - 1)*100, {tauU, 0, 4}, {tauB, 0,

4}, AxesLabel -> {"tU", "bU", "Bias"},

ColorFunction -> ColorData["Bias11"], ColorFunctionScaling -> False,

PlotLegends -> Automatic]

**A2. Results of modifying** $\boldsymbol{b}_{\boldsymbol{U}}$ **in numerical example**

Here we present the 3D graph resulting from the code in Section A1.2.

We observe that as bU (the association between $U$ and $Y$) moves away from 1 (the null), the bias increases in absolute value. As in Figure 2, we see that when tU moves away from the null, bias also increases in absolute value. When either tU or bU is null, there is no selection bias.

**A3. Simulation study R (**[**https://www.r-project.org/**](https://www.r-project.org/)**, version 3.2.0) code**

#Collider bias due to miscarriages

ssize<-10000 #number of pregnancies in time period

lt0<- -1.5 #(to get roughly baseline risk of miscarriage of 0.18)

lb0<- -2.5 #(to get roughly baseline risk of outcome of 0.08)

ltA<- log(3) #log(1), log(2), log(3)

ltU<- log(1.5) #log(1.5), log(2), log(3)

lbU<- log(1.5) #log(1.5), log(2), log(3)

lbA<- log(1.3) #log(1.1), log(1.2), log(1.3)

nS<-1000 #number of simulated datasets

estD<-rep(NA,nS)

varD<-rep(NA,nS)

propsigD<-rep(NA,nS)

propS<-rep(NA,nS)

propY<-rep(NA,nS)

estFull<-rep(NA,nS)

varFull<-rep(NA,nS)

propsigFull<-rep(NA,nS)

set.seed(424434)

for (i in 1:nS){

U<-rbinom(n=ssize,size=1,prob=0.5)

A<-rbinom(n=ssize,size=1,prob=0.6) #about 60% of women on ICS

S<-rbinom(n=ssize,size=1,prob=plogis(lt0+ltA*A+ltU*U)) #risk of miscarriage

Y<-rbinom(n=ssize,size=1,prob=plogis(lb0+lbU*U+lbA*A))

propS[i]<-mean(S==1)

propY[i]<-mean(Y[S==0])

mod<-glm(Y~A,subset=(S==0),family="binomial") #fit model with deliveries only

estD[i]<-coef(mod)[2]

varD[i]<-vcov(mod)[2,2]

propsigD[i]<-(summary(mod)$coefficients[2,4]<0.05)

R<-sample(1:ssize, size=sum(S==0),replace=F)

mod<-glm(Y~A+U,subset=R,family="binomial")

estFull[i]<-coef(mod)[2]

varFull[i]<-vcov(mod)[2,2]

propsigFull[i]<-(summary(mod)$coefficients[2,4]<0.05)

}

mean(propS) # proportion with miscarriages

mean(propY) # outcome rate in delivery cohort

exp(mean(estD))

#quantile(estD,c(0.025,0.975))

mean(propsigD)

exp(mean(estFull))

#quantile(estFull,c(0.025,0.975))

mean(propsigFull)
